# Recombinant *Escherichia coli *produces tailor-made biopolyester granules for applications in fluorescence activated cell sorting: functional display of the mouse interleukin-2 and myelin oligodendrocyte glycoprotein

**DOI:** 10.1186/1472-6750-7-3

**Published:** 2007-01-04

**Authors:** B Thomas Bäckström, Jane A Brockelbank, Bernd HA Rehm

**Affiliations:** 1 Malaghan Institute of Medical Research, Wellington, New Zealand; 2 Institute of Molecular Biosciences, Massey University, Palmerston North, New Zealand

## Abstract

**Background:**

Fluorescence activated cell sorting (FACS) is a powerful technique for the qualitative and quantitative detection of biomolecules used widely in both basic research and clinical diagnostic applications. Beads displaying a specific antigen are used to bind antibodies which are then fluorescently labelled using secondary antibodies. As the individual suspension bead passes through the sensing region of the FACS machine, fluorescent signals are acquired and analysed. Currently, antigens are tediously purified and chemically cross-linked to preformed beads. Purification and coupling of proteins often renders them inactive and they will not be displayed in its native configuration. As an alternative, we genetically engineered *Escherichia coli *to produce biopolyester (polyhdroxyalkanoate=PHA) granules displaying diagnostically relevant antigens in their native conformation and suitable for FACS analysis.

**Results:**

Hybrid genes were constructed, which encode either the mouse interleukin-2 (IL2) or the myelin oligodendrocyte glycoprotein (MOG) fused via an enterokinase site providing linker region to the C terminus of the PHA granule associated protein PhaP, respectively. The hybrid genes were expressed in PHA-accumulating recombinant *E. coli*. MOG and IL2 fusion proteins were abundantly attached to PHA granules and were identified by MALDI-TOF/MS analysis and N terminal sequencing. A more abundant second fusion protein of either MOG or IL2 resulted from an additional N terminal fusion, which did surprisingly not interfere with attachment to PHA granule. PHA granules displaying either IL2 or MOG were used for FACS using monoclonal anti-IL2 or anti-MOG antibodies conjugated to a fluorescent dye. FACS analysis showed significant and specific binding of respective antibodies. Enterokinase treatment of IL2 displaying PHA granules enabled removal of IL2 as monitored by FACS analysis. Mice were immunized with either MOG or OVA (ovalbumin) and the respective sera were analysed using MOG-displaying PHA granules and FACS analysis showing a specific and sensitive detection of antigen-specific antibodies within a wide dynamic range.

**Conclusion:**

*E. coli *can be genetically engineered to produce PHA granules displaying correctly folded eukaryotic proteins and which can be applied as beads in FACS based diagnostics. Since PHA granule formation and protein attachment occurs in one step already inside the bacterial cell, microbial production could be a cheap and efficient alternative to commercial beads.

## Background

Polyhydroxyalkanoate (PHA) granules (biopolyester particles) are formed inside bacterial cells based on the activity and biochemical properties of the PHA synthases and specific biosynthesis enzymes which are involved in PHA precursor supply [1,2]. Biologically, PHA serves as a reserve material. The PHA granule core is composed of PHA and the surface of a phospholipid membrane with embedded or attached proteins. Amphipathic phasin proteins are one group of proteins specifically and hydrophobically interacting with the PHA core (for review see [1,2]). The functional role of the phasins impacting on PHA granule structure has been studied in detail [3-5].

Phasins and their fusion proteins have been increasingly considered for protein production at the PHA granule surface [2,6-8]. Recently, PHA synthase engineering enabled production of the beta-galactosidase and GFP fusion proteins, respectively, at the PHA granule surface [9,10]. The GFP-PHA synthase fusion even enabled monitoring of *in vivo *PHA granule formation indicating that PHA granule formation starts at the cell poles. Only recently, PHA granules have been considered as spherical biopolyester particles which can be stably maintained outside the bacterial cell exerting a size range from about 100 nm to several μm [2,8].

Here we co-expressed the PHA biosynthesis operon from *Cupriavidus necator *with a hybrid gene encoding a phasin fusion protein in *Escherichia coli *in order to mediate the formation of PHA granules efficiently displaying the respective fusion partner. In this study, eukaryotic antigen displaying PHA granules were designed and their application performance with respect to diagnostic applications using fluorescence activated cell sorting (FACS) was evaluated. The displayed myelin oligodendrocyte glycoprotein (MOG) was used as an example depicting diagnostic analysis of the autoimmune disease multiple sclerosis (MS). It was for the first time observed that surface-engineered and antigen displaying PHA granules can be efficiently used for FACS based diagnostics. Thus designed PHA granules, which combine cheap one step production with facilitated folding of proteins, might in future replace commercial beads.

## Results

### Production of PhaP fused to IL2 or MOG at the PHA granule surface

**Figure 1:**
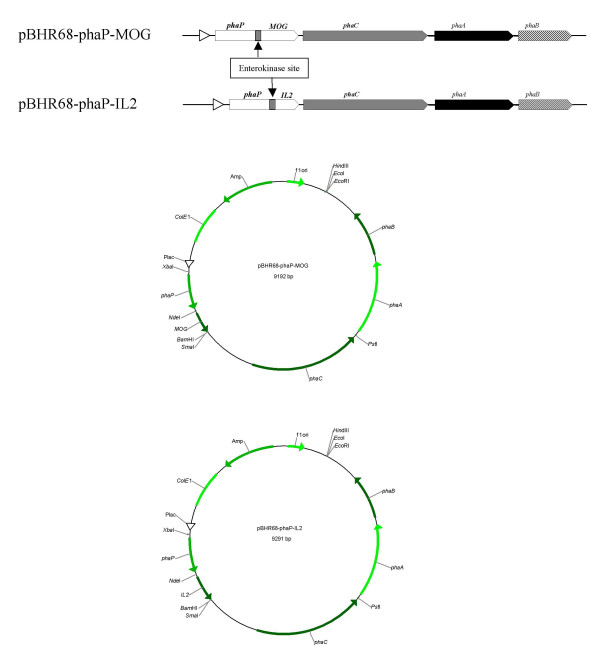
**Schematic view of plasmid constructs mediating production of antigen displaying PHA granules in *E. coli***. Triangle represents the *lac *promoter. *phaP*, phasin gene; IL2, interleukin 2 gene; MOG, myelin oligodendrocyte glycoprotein encoding gene; *phaC*, PHA synthase encoding gene; *phaA*, gene encoding β-kethothiolase; *phaB*, gene encoding acetoacetyl-CoA reductase.

**Figure 2:**
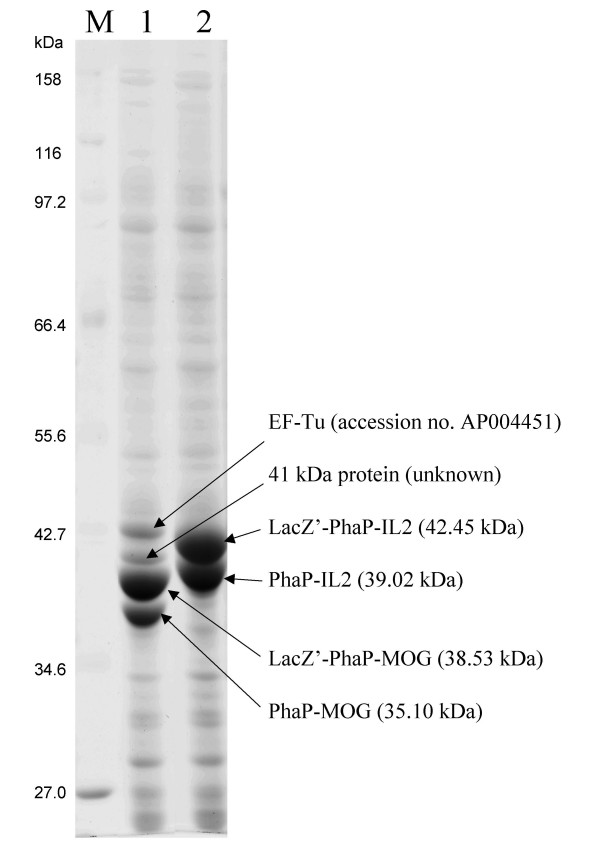
**SDS-PAGE analysis of PHA granule attached proteins**. Proteins were identified by peptide fingerprinting using MALDI-TOF/MS and N terminal amino acid sequencing. LacZ' represents the N terminal 33 amino acid residues of LacZ. M, molecular weight standard; 1, PHA granules isolated from *E. coli *harbouring plasmid pBHR68-phaP-MOG; 2, PHA granules isolated from *E. coli *harbouring plasmid pBHR68-phaP-IL2.

The two immunologically and medically relevant proteins MOG and IL2 derived from *Mus musculus *were each produced as PhaP fusion protein via construction of the respective hybrid gene. The DNA sequences encoding either MOG or IL2 were optimized with respect to the codon usage of *E. coli *and purchased as synthetic DNA fragment [see Additional file 1]. An enterokinase recognition site plus six histidine residues were inserted as linker region between PhaP and IL2 or MOG in order to facilitate independent folding of the fusion partner and to enable specific removal of the antigen from the fusion partner. Plasmids pBHR68-phaP-MOG and pBHR68-phaP-IL2, which both comprise the entire PHB biosynthesis operon in addition to the respective hybrid gene encoding the PhaP fusion protein, were introduced into *E. coli *XL1 Blue (Fig. 1, Table 1). The respective recombinant strains were cultivated and PHA granules isolated. Proteins attached to these granules were separated by SDS-PAGE and prominent proteins were subjected to peptide fingerprinting using MALDI-TOF/MS. Plasmid pBHR68-phaP-MOG mediated the production of PHA granules showing four prominent proteins with apparent molecular weights of 36 kDa, 39 kDa, 41 kDa and 43 kDa (Fig. 2). The 36 kDa and the 39 kDa protein were strongly overproduced and identified as PhaP-MOG fusion proteins using MALDI-TOF/MS analysis (Fig. 2, Table 2). Both proteins were subjected to N terminal amino acid sequencing and the 36 kDa PhaP-MOG fusion showed the expected N terminal amino acid sequence MILTP, whereas the more abundant 39 kDa PhaP-MOG showed an N terminus of MTMITP. The additional two more prominent proteins were subjected to MALDI-TOF/MS analysis and identified the 43 kDa protein as EF-Tu (accession no. AP004451), but no assignment of the 41 kDa could be obtained (data not shown). Plasmid pBHR68-phaP-IL2 mediated the production of PHA granules showing two prominent and strongly overproduced proteins with apparent molecular weights of 39 kDa and 42 kDa. Both proteins were identified as PhaP-IL2 fusion proteins using MALDI-TOF/MS analysis (Table 2) and were subjected to N terminal sequencing and the 39 kDa PhaP-MOG fusion showed the expected N terminus of MILTP, whereas the more abundant 42 kDa PhaP-MOG showed an N terminus of MTMITP.

Batch cultivations led to the production of about 1 × 10^11 ^PHA granules/L cultivation broth corresponding to about 3.0 g protein displayed at the PHA granules surface.

### Native IL2 and MOG proteins were displayed on PHA granules

**Figure 3:**
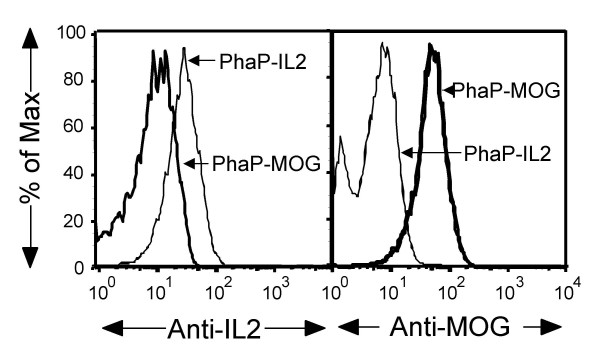
**PHA granules display the native eukaryotic proteins**. PhaP-IL2 and PhaP-MOG granules were produced as described in Materials and Methods. Approximately 5 × 10^8 ^PhaP-IL2 or PhaP-MOG granules were incubated with PC61 (anti-IL2) or 8-18C5 (anti-MOG) antibodies. Samples were collected using a FACsCalibur and then analyzed on FlowJo software. The data were depicted in a "normalized" fashion (% of Maximum). The % of Max is the number of cells in each bin divided by the number of cells in the bin that contains the largest number of cells. One representative experiment of at least three independent experiments, which showed no significant differences, is shown.

To test whether IL2 and MOG were expressed in native form on the surface of the respective PHA granules, antibodies that recognize correctly folded IL2 (PC61) or MOG (8-18C5) and FACS technology were used [11,12]. The size of PHA granules produced in *E. coli *has been reported to be about 100–500 nm [1], and were detected in the forward scatter (FSC) and 90° side scatter (SSC) detectors set to list mode at a 256-channel resolution (data not shown). PHA granules were incubated with unlabelled mouse anti-MOG antibodies, followed by APC-labelled anti-mouse IgG antibodies, or with PE-labelled anti-IL2 antibodies. The fluorescent intensity was then measured using FACS technology. Results show that the monoclonal antibodies specifically recognized PHA granules displaying the specific antigen, but not an un-related antigen (Fig. 3). Both PC61 and 8-18C5 recognize only the respective native protein indicating that correctly folded IL-2 or MOG proteins were formed at the surface of the respective PHA granules. These PHA granules showed consistent performance in FACS analysis at least one year when stored at 4°C.

### PhaP-IL2 and PhaP-MOG fusion proteins containing the Asp-Asp-Asp-Asp-Lys recognition sequence are cleavable with enterokinase

**Figure 4:**
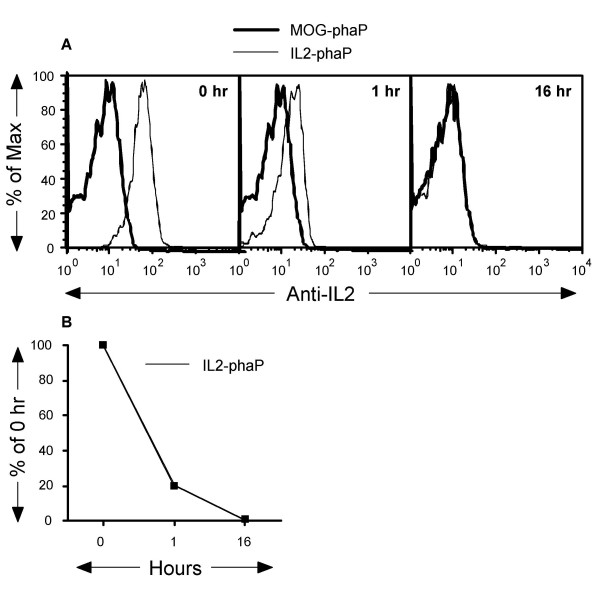
**Enterokinase treatment of antigen displaying PHA granules and FACS analysis**. PhaP-IL2 and PhaP-MOG granules were incubated with enterokinase for the indicated period of time, washed and then tested for the amount of native proteins remaining at the surface of the granules. In (A), the anti-IL2 PC61 and anti-MOG 8-18C5 monoclonal antibodies were used for this purpose as described for Figure 1. In (B), the percentage of IL2 protein detected after 1 and 16 hours incubation with enterokinase is shown. The data was depicted in a "normalized" fashion (% of Max). One representative experiment of at least three performed experiments, which showed no significant differences, is depicted. After overnight incubation no detectable staining using fluorescent anti-IL2 antibodies could be observed.

To enable removal and/or purification of recombinant eukaryotic proteins, which form inactive inclusion bodies in *E. coli*, an enterokinase recognition sequence encoding for the Asp-Asp-Asp-Asp-Lys peptide was incorporated immediately downstream of PhaP. To determine if the recognition sequence was readily accessible, PhaP-IL2 and PhaP-MOG containing PHA granules were incubated for 16 h with enterokinase. Samples of PhaP-IL2 and PhaP-MOG granules were removed before, after 1 h and after 16 h of incubation with enterokinase to determine the level of native protein remaining at the surface of the respective PHA granule. PhaP-IL2 and PhaP-MOG granules were incubated with PE-conjugated anti-IL2 and samples analysed by FACS (Fig. 4A). A similar pattern of decreased protein levels after incubation with enterokinase was found with PhaP-MOG granules incubated with anti-MOG monoclonal antibodies (data not shown). The level of IL2 was reduced by ~80% after 1 h incubation and after 16 h incubation no significant levels of IL2 protein were detectable at the surface of the granules (Figure 4B). Overall, results shown in Figures 3 and 4 demonstrate that correctly folded recombinant proteins can be expressed as a fusion protein together with PhaP and that the fusion partner can be completely removed by enterokinase treatment.

### PHA granules displaying PhaP-MOG fusion proteins were used in FACS-based assays to detect antigen-specific serum antibodies

**Figure 5:**
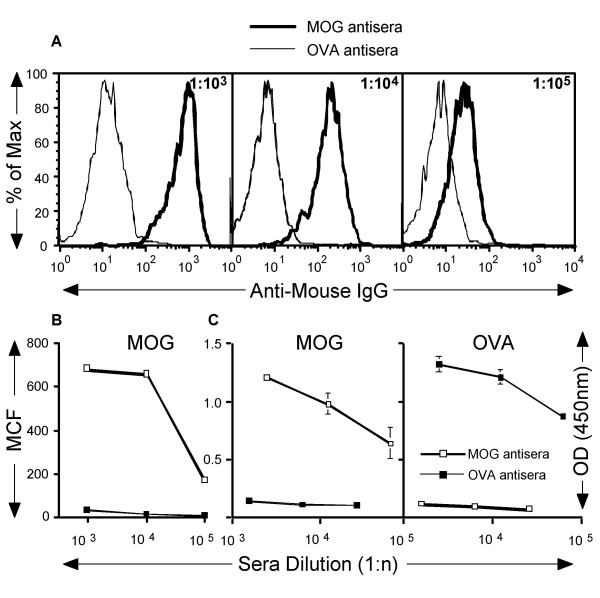
**FACS-based detection of antigen-specific antibodies using antigen displaying PHA granules**. In (A), MOG-phaP granules were incubated with serial dilutions of pooled antisera from five MOG or OVA immunized mice. Granules were extensively washed and then incubated with biotinylated anti-mouse IgG, followed by PE-conjugated streptavidin. The data was depicted in a "normalized" fashion (% of Max). One representative experiment, of at least two experiments performed, is depicted. In (B), the mean channel fluorescent for each dilution of antisera from MOG or OVA immunized mice binding to MOG-phaP was measured and depicted. In (C), ELISA was performed on anti-sera from MOG- and OVA-immunized mice. Serial dilutions of the anti-sera were added to MOG- or OVA-coated wells and incubated for 30 minutes. Biotinylated anti-mouse IgG was then added to the washed wells, followed by HRP-conjugated streptavidin and TMB substrate. The optical density was read at 450 nm and the results from one representative experiment, of at least two experiments performed, are shown. In (C), data are displayed as mean ± SEM from triplicate samples.

Since PHA granules can be detected using FACS technology, we tested whether PhaP-MOG granules could be used to detect and quantify the level of antigen-specific antibodies from immunized animals. For this purpose, mice were immunized with recombinant MOG or OVA protein in CFA to induce an antibody response to MOG and OVA, respectively. Twenty-eight days post immunization, mice were tail-bled and immune sera collected. Sera from five different mice were pooled and tested for binding to PhaP-MOG granules. Results in Figure 5A showed that MOG-immunized, but not OVA-immunized, anti-sera contained antigen-specific IgG antibodies that recognized the PhaP-MOG fusion protein. The sera could be diluted >1:10^5 ^fold and still showed a significant binding of IgG to the PhaP-MOG granules (Figure 5B). Sera from OVA-immunized mice did not bind significantly to PhaP-MOG granules. However, OVA immunized mice produced OVA-specific IgG, as micro-titre wells coated with OVA, but not with MOG, showed bound IgG which was easily detectable using ELISA (Figure 5C). These results illustrate that recombinant eukaryotic proteins displayed with PhaP as a fusion protein can be applied using FACS technology to detect and quantify the level of antigen-specific antibodies from vaccinated animals.

## Discussion

**Figure 6:**
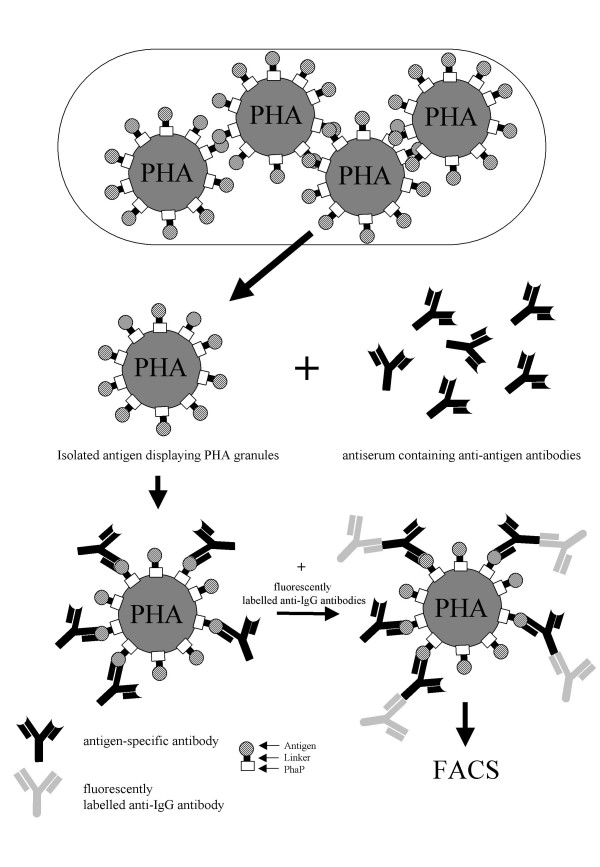
Schematic view of the microbial production of antigen displaying PHA granules and their use in FACS analysis

In this study, the surface of PHA granules produced by recombinant *E. coli *was subjected to protein engineering to display natively folded eukaryotic proteins, which serve as antigens in diagnostics (Fig. 6). Both chosen eukaryotic proteins MOG and the cytokine IL-2 form inclusion bodies upon production in *E. coli *[13,14]. A microbial protein production system avoiding the formation of inclusion bodies would have a huge advantage by circumventing the tedious process of solubilization and refolding. The PHA granule attached structural protein PhaP was N terminally fused to either MOG or IL2 (Fig. 1). A strong overproduction under *lac *promoter control of the respective fusion proteins could be observed (Fig. 2). The fusion proteins could be identified by peptide fingerprinting using MALDI-TOF/MS analysis and by N terminal amino acid sequencing. Interestingly, the fusion proteins appeared in two versions with different apparent molecular weight. The smaller molecular weight version of the fusion protein showed the theoretical molecular weight of the respective fusion protein. The N terminal sequence (MTMITP) of the larger molecular weight version suggested that the N terminal 33 amino acid residues (theoretical molecular weight of 3.4 kDa) of LacZ were fused to the N terminus of PhaP fusion protein based on start codon upstream of the start codon of the hybrid gene encoding the PhaP-MOG or PhaP-IL2. This was also in accordance with the apparent molecular weight difference of 3.5 kDa between the two versions. Interestingly, the LacZ'-PhaP-MOG/IL2 fusion proteins were more abundant than the respective single fusions (Fig. 2). These data suggest that N terminal fusions to PhaP do not interfere with the binding to the PHA core of PHA granules and that the copy numbers can be even enhanced. This is unexpected considering the current phasin protein model [5]. The presence of the two *E. coli *proteins 41 kDa and EF-Tu at the MOG displaying PHA granule surface might be due to an unspecific interaction with PHA granule surface, which has been already observed elsewhere [10]. In contrast to a previous study, where multiple copies of PhaP were required for binding of prokaryotic protein fusions, a single copy was sufficient using an enterokinase site linker and eukaryotic proteins [6].

Engineered PHA granules were subjected to FACS analysis using antibodies specifically recognizing the respective native protein folds, which showed the display of the natively folded eukaryotic proteins MOG and IL2 at the PHA granule surface as well as the applicability of designed PHA granules for FACS-based diagnostics (Fig. 3). Although MOG and IL2 are secreted proteins, the derived protein domains were properly folded attached to the PHA granule in the reducing cytosol of *E. coli *while avoiding the formation of protein inclusion bodies. Although *E. coli *strains (e.g. Origami) are available, which provide an oxidative cytosol, protein overproduction often leads to inclusion body formation. Enterokinase treatment of MOG or IL2 displaying PHA granules and the complete release of the antigen suggested that the respective antigen was exposed at the PHA granule surface (Fig. 4). This surface exposure enables efficient antigen specific antibody binding. To evaluate the suitability of MOG or IL2 displaying PHA granules for qualitative and quantitative FACS-based antibody detection, mice were immunized with MOG or OVA (control) and induction of specific antibody production was assessed and confirmed by ELISA (Fig. 5). These MOG or OVA antisera were analysed using the MOG displaying PHA granules and FACS technology, which clearly indicated that anti-MOG antibodies can be specifically detected at least up to an antisera dilution of 1:100,000 (Fig. 5). In this study, it was demonstrated that eukaryotic proteins can be functionally displayed at the PHA granule surface using protein engineering of PhaP. Evidence was provided that the fusion proteins (antigens) were exposed at the PHA granule and thus PHA granules could be used to capture antibodies. This feature in combination with the particle properties of the PHA granules led to an outstanding performance in FACS based diagnostics, particularly considering the signal to noise ratio and dynamic range of antibody detection. It was recently shown that ZZ domain displaying PHA granules, which were produced via protein engineering of the PHA synthase, were suitable for IgG purification [15]. When compared to commercial beads, engineered PHA granules showed increased sensitivity with similar distribution of signal intensities in FACS. Overall, protein engineering of PHA granule surface proteins provides a novel molecular tool for the display of antigens for FACS-based diagnostics.

## Conclusion

In this study, it was demonstrated that correctly folded eukaryotic proteins can be abundantly produced at the PHA granule surface as phasin fusion proteins. Isolated PHA granules displaying the respective eukaryotic proteins could be used as beads for specific and sensitive antibody detection using FACS technology. These native antigen displaying PHA granules were manufactured by recombinant *E. coli *without the need of antigen purification and chemical cross-linking to independently produced beads. Often purification of these proteins requires tedious refolding at low efficiency. The production of functional eukaryotic proteins at the PHA granule surface represents a novel *in vivo *matrix-assisted protein folding system avoiding aggregation of protein folding intermediates. Moreover, PHA granules could be stored for at least one year at 4°C without loss of performance in FACS supporting their potential use in diagnostic applications.

This work opens up an alternative route for the production of protein displaying beads harnessing nature's capacity to produce spherical polymer beads which surface can be functionalized by engineering of specifically bead associating proteins (Fig. 6). Multiple functionality might be easy achievable by co-expression of various hybrid genes suggesting that this biotechnological bead production strategy might represent a versatile platform technology.

## Methods

### Bacterial strains and growth conditions

Bacterial strains, plasmids and oligonucleotides used in this study are listed in Table 1. *Cupriavidus necator *and *E. coli *XL1 Blue were grown at 37°C. When required, ampicillin 75 μg/ml was added. All chemicals were purchased from Sigma-Aldrich (St. Louis, Mo., USA).

### Isolation, analysis and manipulation of DNA

General cloning procedures and isolation of genomic DNA were performed as described elsewhere [19]. DNA primers, deoxynucleoside triphosphate, *Taq *and Platinum *Pfx *polymerases were purchased from Invitrogen™ (CA, USA). DNA sequences of new plasmid constructs were confirmed by DNA sequencing according to the chain termination method using the model ABI310 automatic sequencer. Plasmids used in this study are listed in Table 1.

### Construction of plasmids mediating production of either IL2 or MOG displaying PHA granules

The DNA fragments encoding either the full length mature IL2 protein (amino acids 60–169, accession no. AAN38301) or the extracellular part of the MOG protein (amino acids 1–117, accession no. Q61885) from mouse were synthesized by GenScript Corp. (USA). The codon usage was optimized for expression in *E. coli *[see Additional file 1]. Each DNA fragment contained an *Nde*I site at the 5' end and a *Bam*HI site at the 3' end. These DNA fragment were inserted into the *Sma*I site of pUC57 directly after synthesis by GenScript Corp. (USA) resulting in plasmids pUC57-MOG or pUC57-IL2, respectively. The coding region of *phaP1 *gene was amplified by PCR from chromosomal DNA of *C. necator *using oligonucleotides phaP-XbaI-NdeI and phaP-NdeI listed in Table 1 and introducing an *Xba*I site at the 5' end and an *Nde*I site including an enterokinase site at the 3' end. The PCR product was subcloned into TA cloning plasmid pCRII. The *phaP *coding region was again amplified from plasmid pCR-phaP using oligonucleotides phaP-XbaI including an *E. coli *ribosomal binding site and phaP-NdeI. The resulting PCR product was subcloned into the *Xba*I and *Nde*I sites of pHAS.

Either pUC57-MOG or pUC57-IL2 was hydrolyzed with *Nde*I and *Bam*HI and the corresponding DNA fragments were subcloned into pHAS-phaP resulting in plasmids pHAS-phaP-MOG and pHAS-phaP-IL2, respectively. The respective fusion protein encoding region was then subcloned into pBHR68 using *Xba*I and *Bam*HI sites downstream of the *lac *promoter and upstream of the PHB biosynthesis operon (Fig. 1).

### Production of phasin fusion proteins at the PHA granule surface

Cells of *E. coli *XL1 Blue were transformed with plasmids pBHR68-PhaP-IL2 and pBHR68-PhaP-MOG, respectively. Transformants were grown at 37°C and induced by adding isopropyl-β-D-thiogalactopyranoside (IPTG) to a final concentration of 1 mM. After growth for 48 h, cells were harvested by centrifugation and subjected to PHA granule isolation.

### Isolation of PHA granules

Cells were harvested by centrifugation for 15 min at 5,000 × g and 4°C. The sediment was washed and suspended in 3 volumes of 50 mM phosphate buffer (pH7.5). Cells were passed through French Press four times at 8000 psi. The cell lysate (0.75 ml) was loaded onto a glycerol gradient (88 % and 44 % (v/v) glycerol in phosphate buffer). After ultracentrifugation for 2.5 h at 100,000 × g and 10°C, granules could be isolated from a white layer above the 88 % glycerol layer. The PHA granules were washed with 10 volumes phosphate buffer (50 mM, pH7.5) and centrifuged at 100,000 × g for 30 min at 4°C. The sediment containing the PHA granules was suspended in phosphate buffer and stored at 4°C.

### Sodium dodecyl sulfate (SDS) polyacrylamide gel electrophoresis (PAGE)

Protein samples were routinely analyzed by SDS-PAGE as described elsewhere [20]. The gels were stained with Coomassie brilliant blue G250. Protein bands of interest were cut off the gel and analyzed by matrix assisted laser desorption/ionization time-of flight mass spectrometry (MALDI TOF-MS).

### MALDI-TOF mass spectrometry

Mass spectrometric analyses of tryptic peptides were carried out on a MALDI VOYAGER DE-PRO time of flight mass spectrometer from PerSeptive BioSystems (Framingham, MA) utilizing a nitrogen laser, emitting at 337 nm, and an accelerating voltage of 25 kV. Measurements were performed in the delayed extraction mode using a low mass gate of 2000. The mass spectrometer was used in the positive ion detection and linear mode. Samples of the digestion mixture were placed directly on a 100-position sample plate, and allowed to air-dry after the addition of an equal volume of saturated solution of 3,5-dimethoxy-4-hydroxycinnaminic acid (sinapinic acid) in 50% acetonitrile and 0.3% TFA.

### Mice

C57BL/6 mice were originally purchased from the Jackson Laboratory (Bar Harbor, ME). All mice were maintained by the Biomedical Research Unit, Malaghan Institute of Medical Research, Wellington, New Zealand. Experimental protocols were approved by the Victoria University of Wellington Animal Ethics Committees and performed according to the guidelines of their guidelines. In all experiments, sex and age matched mice were used between 8–12 weeks of age.

### Mouse serum IgG responses

C57BL/6 mice were immunized with 150 μg recombinant mouse MOG protein consisting of amino acid 1–117 (MOG_1–117_) of the matured protein [21] or 150 μg ovalbumin protein (OVA, Sigma-Aldrich) emulsified in Complete Freund's adjuvant (CFA), containing 500 μg *Mycobacterium tuberculosis *(both DIFCO Laboratories). The emulsion (100 μl) was injected subcutaneously over the flanks. Blood was collected from tail bleeds 28 days post-immunization, centrifuged at 13,000 g for 1 min, and then the top layer of serum was removed and stored at -20°C. Serum was tested for antigen-specific total IgG, using the PhaP-MOG granules and flow cytometry or ELISA (see below).

### Cleavage of IL2 from IL2 displaying PHA granules

The enterokinase recognition sequence Asp-Asp-Asp-Asp-Lys was introduced between PhaP and IL-2 to enable cleavage of the IL-2 protein from the granules. Twenty-five μg of bovine enterokinase (Sigma-Aldrich, E-5144) in 10 mM Tris/HCl, 10 mM CaCl_2 _(pH8) were incubated with 2 × 10^10 ^PhaP-IL2 or PhaP-MOG granules at 37°C, and at indicated time points samples were removed, washed with PBS, and stored at 4°C until use. The relative amount of native IL2 and MOG at the surface of the granules was determined using flow cytometry.

### Flow cytometry

PhaP-MOG or PhaP-IL2 granules (~5 × 10^8^/well) were added to 96-well round-bottom plates (Becton, Dickinson and Company) and washed twice with FACS-buffer (PBS, 1% Foetal calf serum, 0.1% sodium azide, 5 mM EDTA, pH8). IL2-phaP or MOG-phaP granules were then incubated with phycoerythrin conjugated anti-IL2 monoclonal antibody (clone PC61, PharMingen) or a mouse anti-MOG monoclonal antibody (clone 8.18-C5, kindly provided by C. Bernard). Following 15 minutes incubation at ambient temperature, granules were washed twice in fluorescence activated cell sorting (FACS) buffer. Allophycocyanin-conjugated anti-mouse IgG (Jackson ImmunoResearch Laboratories Inc.) polyclonal antibodies were added to the MOG displaying PHA granules and incubated for 15 minutes, and then washed twice. At least 10,000 events for each sample were collected and analysed, using a BD FACsCAlibur and FlowJo analysis software (Tree Star, San Carlos, CA, USA). For detection of MOG-specific antibody responses, sera from MOG or OVA immunized mice were diluted in FACS-buffer and then incubated for 30 minutes at ambient temperature with MOG-phaP granules. The granules were then washed and incubated with biotinylated anti-mouse total IgG, followed by PE-conjugated streptavidin. At least 10,000 events for each sample were collected and analysed on a BD FACsCAlibur and FlowJo software (Tree Star).

### ELISA

Round-bottom 96-well plates (Becton, Dickinson and Company) were coated overnight at 4°C (50 μl/well) with 3 μg/ml of recombinant mouse MOG_1–117_or OVA protein (Sigma-Aldrich) in PBS. Supernatant was discarded and wells blocked by adding 1% BSA in PBS (100 μl/well) for 1 h at ambient temperature. Plates were then wash 3 times with 10 mM Tris/HCl, pH 7.5, 0.05% Tween 20 (ELISA buffer). Diluted sera (50 μl/well) in 0.1% BSA/PBS from MOG_1–117 _or OVA immunized mice were added and after 2 h, plates were washed 3 times with ELISA buffer. Biotin-conjugated anti-mouse IgG antibodies (Southern Biotechnology, Ltd) diluted 1:4,000 in 0.1% BSA/PBS were added for 1 h, and wells then wash 3 times with ELISA buffer. Amdex Streptavidin-HRP (Amersham Biosciences) diluted 1:3,000 in 0.1% BSA/PBS, was added to each well (50 μl/well) and incubated for 30 minutes. Plates were then wash 3 times and 3,3',5,5'-tetramethylbenzidine (TMB) added for 5–30 minutes. Colour development was stopped using 2 M H_2_SO_4 _and the ELISA plates were then read at 450 nm on a Benchmark microplate reader (Bio-Rad Laboratories Inc. Hercules, CA, USA).

## Authors' contributions

TB designed and conducted all immunological experiments including FACS analysis and identification of the antigens to be used as examples. TB wrote a part of the manuscript. JB carried out all cloning, PHA granule production/isolation and SDS-PAGE analysis. BR conceived the experimental strategy for the production of the engineered PHA granules and their analysis as well as wrote the manuscript. All authors read and approved the final manuscript.

## Supplementary Material

Additional File 1 DNA sequence of synthetic DNA fragments encoding either MOG or IL2 with optimized codon usage for expression in *E. coli*. Click here for file

## Figures and Tables

**Table 1:** Bacterial strains, plasmids and oligonucleotides used in this study

Strains	Genotype	Source or reference
*Escherichia coli*	*recA1*, *endA1*, *gyrA96*, *thi-1*, *hsdR17 *(r_k_^-^, m_k_^+^),	[16]
XL1-Blue	*supE44*, *relA1*, -, *lac *[F', *proAB, lacI*^*q*^, *lacZ*Δ*M15*, Tn*10*(Tc^r^)]	
*Cupriavidus necator *H16	Wildtype (PHA^+^)	DSMZ 428
Plasmids		
pCRII	Amp, ColE1 origin	Initrogen
pBHR68	PHB biosynthesis operon from *C. necator *in pBluescriptSK^-^	[17]
pHAS	pET-14b containing *Nde*I/*Bam*HI inserted *phaC *gene from *C. necator*	[18]
pUC57-MOG	DNA fragment encoding N terminal 1–173 amino acids of MOG from mouse inserted in *SmaI *site of pUC57	This study
pUC57-IL2	DNA fragment encoding N terminal 60–169 amino acids of IL2 from mouse inserted in *SmaI *site of pUC57	This study
pCR-phaP	pCRII containing *phaP *gene from *C. necator*	This study
pHAS-phaP	pHAS containing the *phaP *gene inserted into *Xba*I/*Nde*I sites	This study
pHAS-phaP-MOG	pHAS-phaP containing the MOG encoding DNA fragment inserted into *Nde*I/*Bam*HI sites	This study
pHAS-phaP-MOG	pHAS-phaP containing the IL2 encoding DNA fragment inserted into *Nde*I/*Bam*HI sites	This study
pUC57-phaP-MOG	DNA fragment encoding PhaP inserted into *Xba*I and *Nde*I site of plasmid pUC57-MOG	This study
pUC57-phaP-IL2	DNA fragment encoding PhaP inserted into *Xba*I and *Nde*I site of plasmid pUC57-IL2	This study
pBHR68-phaP-MOG	DNA fragment encoding PhaP-MOG fusion protein subcloned from pUC57-phaP-MOG via *Xba*I and *Bam*HI into pBHR68	This study
pBHR68-phaP-IL2	DNA fragment encoding PhaP-IL2 fusion protein subcloned from pUC57-phaP-IL2 via *Xba*I and *Bam*HI into pBHR68	This study
Oligonucleotides		
phaP-*Xba*I-*Nde*I	5'-aaaaatctagaaaaaggagatatacatatgatcctc-accccggaacaag-3'	This study
phaP-*Xba*I	5'-aaaaatctagaaaaaggagatatacgtatgatcctc-accccggaacaag-3'	This study
phaP-*Nde*I	5'-aaaaacatatggtggtgatggtgatgcgagccgcgt-ttatcatcatcatcggcagccgtcgtcttc-3'	This study

**Table 2:** Identified peptide fragments of proteins analyzed by MALDI-TOF/MS

protein	peptide fragments
LacZ'-PhaP-IL2	A91-Y120, E96-A114, E121-E135, E190-T218,
PhaP-IL2	A57-Y86, E62-A80, E87-E101, E156-T184, L300-G320
LacZ'-PhaP-MOG	N15-M34, W18-M34, L89-H118, A94-H118, L119-N155, L113-V133, A156-S169, T185-T216, A188-T216, T222-G232, G247-L255, T273-P283, V289-R308
PhaP-MOG	L55-H84, A60-H84, L85-N121, L79-V99, A122-S135, A154-T182, A173-T188, G247-L255, T273-P283, T275-V288, V289-R308.
