# Supplementary material for: Recombinant Escherichia coli produces tailor-made biopolyester granules for applications in fluorescence activated cell sorting: functional display of the mouse interleukin-2 and myelin oligodendrocyte glycoprotein
Source: BMC Biotechnol. 2007 Jan 4;7:3. doi: 10.1186/1472-6750-7-3 (PMC1781935; doi:10.1186/1472-6750-7-3)
Supplement: Additional File 1 — DNA sequence of synthetic DNA fragments encoding either MOG or IL2 with optimized codon usage for expression in E. coli. [file 1472-6750-7-3-S1.doc]

**Additional files 1**

**DNA sequence of IL2 encoding DNA fragment**

**Optimized Sequence: Length: 468, GC%:48.93, Minimum Free Energy:-149.54**

TCTAGACATATGGCACCAACGTCCAGCTCGACCAGCAGTTCTACCGCAGAAGCCCAGCAG

CAGCAGCAGCAGCAGCAACAGCAGCAGCAACATCTGGAACAGCTGCTGATGGATCTGCAG

GAACTGCTGAGTCGTATGGAAAATTATCGTAATCTGAAACTGCCACGTATGCTGACGTTT

AAATTCTATCTGCCAAAACAGGCTACGGAACTGAAAGATCTGCAGTGCCTGGAAGATGAA

CTGGGTCCACTGCGTCACGTCCTGGATCTGACCCAGTCCAAATCCTTTCAGCTGGAAGAC

GCTGAAAATTTTATCTCCAATATCCGTGTGACGGTGGTGAAACTGAAAGGTTCTGACAAC

ACGTTTGAATGCCAATTTGATGACGAATCAGCTACCGTGGTCGATTTTCTGCGTCGTTGG

ATCGCCTTTTGCCAGTCGATTATTTCGACAAGCCCACAGTAAGGATCC

**DNA sequence of MOG encoding DNA fragment**

**Optimized Sequence: Length: 372, GC%:48.12, Minimum Free Energy:-110.90**

TCTAGACATATGGGTCAGTTTCGTGTGATTGGCCCAGGTTATCCAATCCGTGCACTGGTA

GGTGATGAGGCAGAACTGCCATGTCGTATTTCCCCAGGCAAAAATGCTACCGGTATGGAA

GTAGGTTGGTATCGTTCCCCATTTTCCCGTGTGGTGCATCTGTATCGTAACGGCAAAGAT

CAGGATGCCGAACAGGCCCCAGAATATCGTGGTCGTACAGAGCTGCTGAAAGAAACGATT

AGCGAAGGCAAAGTTACTCTGCGTATTCAGAATGTGCGTTTTAGCGATGAAGGTGGCTAT

ACCTGTTTCTTCCGTGATCACTCTTACCAGGAAGAAGCCGCCATGGAACTGAAAGTTGAA

GATTAAGGATCC
